# Popular Science Department

**Published:** 1882-11

**Authors:** 


### POPULAR SCIENCE DEPARTMENT.

### THE ORIGIN OF MUSIC.

In the theory of Helmholtz, music expresses the different dispositions of the soul by imitating the characteristic particularities of movement in space, and by thus translating the forces and impulses that produce the movement. While he admits that it may have been at first only an imitation of the instinctive modulations of the voice corresponding with the different states of the mind, he does not consider this fact contradictory to his definition for the natural processes of vocal expression are capable of being traced back to the same elements. "Rhythm and accentuation express directly the rapidity and vivacity of corresponding psychical movements; a vehement effort causes the voice to rise; the desire to produce an agreeable impression on another person prompts us to select a pleasant tone; and thus the efforts to imitate the involuntary modulations of the voice, to enrich and make more expressive the recitation of words, may very probably have guided our ancestors in seeking out the means for musical expressions."

This is probably the real origin of music; and it is in this direction that we should look in investigating its nature.

Two elements closely connected, but quite different and having each its peculiar function, may be distinguished in the analysis of spoken language—the intonation and the articulation of the emitted sound. No doubt they are the interpreters of the two great human faculties of intelligence and feeling. Speech, then, is a complex physiological resultant, the double image of a double inner condition. The elements it represents cannot be conceived as isolated from each other, any more than we can conceive a human organization a pure intelligence. We all know the important part intonation plays in conversation, and how by it the general sense, the whole expression of the spoken words, may be varied indefinitely.

Having thus found the origin of music in the imitation of these nstinctive modulations of speech, it should be easy to draw from this an exact idea of its nature ; for, without doubt, to read verse well, to declaim with warmth and conviction, is only to perform in advance the work of the musician. We have now a whole class of musical phrases which are only exaggerations of spoken intonations ; they are our recitatives. The music of uncultivated peoples is mostly recitative ; so also was a large part of the music of the middle ages. The rules and grammar of music and its particular features are the growth of modern times.

This conception of music as the language of sensibility permits us to explain the characteristic features of its action. We must consider, first, that this language, like its twin sister the language of ideas, has suffered a progressive evolution, and has with us reached a great perfection, and consequently a great complexity in its laws and processes. Among primitive peoples of few ideas, whose feelings show few variations of shade in expression, music is almost wholly confined to a few modulations expressive of the principal divisions of feeling—love, joy, sorrow and warlike ardor. Civilization, with its refinements, has produced a music that has grown constantly richer in shades and means of expression, to the point which has been reached by the great masters of our age.—M. HERICOURT, *in Popular Science Monthly for December.*

### ANNULAR ECLIPSE OF THE SUN.

On the 10th of November there will be an annular eclipse of the sun, invisible in this country, but visible in the Southern Pacific

Ocean. The path of the eclipse lies wholly in the Pacific Ocean, commencing a little east of the Island of Celebes, including several small islands in the vicinity, the southern part of New Guinea, the whole of New Caledonia, and a few small islands scattered along the route. The rest of the track is over a boundless waste of waters. To observers on these islands, and to those who chance to be on the ocean track at the time, the sun will present the appearance of a ring of dazzling light surrounding the moon's intervening disk. An annular eclipse ranks next to a total eclipse as a spectacle of surpassing beauty, though it is far from being as awe-inspiring or as important to the interests of science.

The difference between total and annular eclipses is easily comprehended. The positions and apparent magnitudes of the sun and moon are constantly varying. When, at new moon, the center of the moon happens to pass directly over the center of the sun; if, at the same time, the sun is at or near his greatest distance from the earth, and the moon is at or near her least distance from the earth, the apparent diameter of the moon will exceed that of the sun, and the sun will be entirely hidden from view. There will then be a total eclipse of the sun, visible to observers near the line joining the centers of the sun and moon. These conditions occurred on the 17th of May, when a total solar eclipse took place. The diameter of the sun at that time was 31′ 41·6″. The diameter of the moon was 31′ 43·8″, exceeding that of the sun 2·2″, sufficient to produce a total eclipse.

When, at new moon the center of the moon happens to pass directly over the center of the sun; if, at the same time, the sun is at or near his least distance from the earth, and the moon is at or near her greatest distance from the earth, the apparent diameter of the moon will be less than that of the sun, and it is evident that the whole surface of the sun cannot be obscured. There will then be an annular eclipse—called so from the Latin word annulus, meaning a ring—which will be visible to observers near the line joining the centers of the sun and moon. Such a combination will occur on the 10th of November. The diameter of the sun will then be 32′ 24·2″. The diameter of the moon will be 30′ 6·6″, which is 2′ 17·6″ less than that of the sun. A narrow ring of light will therefore appear encircling the darkened center.

The present year numbers but two eclipses on its annals. There have been no eclipses of the moon, and the two solar eclipses are those to which we have referred. But the rare event of the transit of Venus, which will take place on the 6th of December, deserves to be numbered with the solar eclipses, for it is due to a similar cause. The planet obscures as much of the solar disk as she is capable of doing, when she passes like a black point over his disk. If she were as near as the moon she would cause an eclipse of the sun that would last long enough to be of great assistance in the solution of many vexed problems concerning solar physics.

If the eclipses of the year are few in quantity they make up for the deficiency in their excellent quality.

A total solar eclipse, an annular eclipse, and a transit of Venus are seldom the sole records on the annals of a single year. It is much to be regretted that the path of the coming annular eclipse falls upon a portion of the world where there will be few to look upon the superb spectacle when the moon hides the sun's face with the exception of a narrow ring of dazzling light.—*Scientific American.*

### THE FUTURE OF GAS.

Gas is an institution of the utmost value to the artisan ; it requires hardly any attention, is supplied upon regulated terms, and gives with what should be a cheerful light a genial warmth, which often saves the lighting of a fire. The time is, moreover, not far distant, I venture to think, when both rich and poor will largely resort to gas as the most convenient, the cleanest, and the cheapest of heating agents, and when raw coal will be seen only at the colliery or the gas-works. In all cases where the town to be supplied is within say thirty miles of the colliery, the gas-works may with advantage be planted at the mouth, or still better at the bottom of the pit, whereby all haulage of fuel would be avoided, and the gas, in its ascent from the bottom of the colliery, would acquire an onward pressure sufficient probably to impel it to its destination. The possibility of transporting combustible gas through pipes for such a distance has been proved at Pittsburg, where natural gas from the oil district is used in large quantities.

The *quasi* monopoly so long enjoyed by gas companies has had the inevitable effect of checking progress. The gas being supplied by meter, it has been seemingly to the advantage of the companies to give merely the prescribed illuminating power, and to discourage the invention of economical burners, in order that the consumption might reach a maximum. The application of gas for heating purposes has not been encouraged, and is still made difficult, in consequence of the objectionable practice of reducing the pressure in the mains during day-time to the lowest possible point consistent with prevention of atmospheric indraught. The introduction of the electric light has convinced gas managers and directors that such a policy is no longer tenable, but must give way to one of technical progress ; new processes for cheapening the production and increasing the purity and illuminating power of gas are being fully discussed before the Gas Institute ; and improved burners, rivaling the electric light in brilliancy, greet our eyes as we pass along our principal thoroughfares.—C. WILLIAM SIEMENS, F. R. S., *in Popular Science Monthly for December.*

### MASTODON SKELETON FOUND.

While making a cut for the extension of the Kentucky Central Railroad on Joseph Mitcher's farm, last Saturday, Thomas M. Farrow, section foreman, and his gang of hands unearthed the skeleton of a gigantic prehistoric animal, supposed to be a mastodon, but its immense size is larger than the remains even of that huge animal of which we ever heard of being found. At any rate the animal belongs to a species long extinct, and entirely unknown to the post-di-diluvian world. Mr. Farrow, when he discovered the skeleton, had the earth removed from above it carefully as possible, and made the following measurements, which he has kindly furnished us :

Length of animal, 40 feet.
Height, 23 feet 8 inches.
Length of head, 15 feet 4 inches.
Across the knee, 18½ inches.
One bone 10¼ inches in diameter, and another 8 inches in diameter.
Leg from shoulder blade to ankle, 14 feet 7 inches.
End at ankle bone, 7 inches in diameter.
Knee socket, 2 feet 1½ inches in diameter.
Shoulder blade, 3 feet 5 inches wide.
Jaw-bone, 15 inches in diameter.
Bone from ankle to knee, 3 feet 8 inches in length.

One tooth 3 inches wide and 5½ inches long and three teeth 13 inches.

The skeleton position was on a ledge of rock, embedded in a mass of tough blue clay, above which was a stratum of yellow clay, covered by about two feet of soil, the whole forming a thickness of some seven feet. The manner in which the teeth are worn indicates the monster to have been of a herbivorous species, and it is not unlikely, from the position in which the bones were found, that the animal got bogged in the bed of tough mud, fell down in its struggles to free itself, and being too unwieldly to arise, perished there. A blast hole charged with a keg and a half of powder had been sunk near the skeleton, which was greatly shattered by the force of the explosion ; otherwise Mr. Farrow could have had it taken out more perfectly. Some of the bones are in good state of preservation, but the major portion of them crumbled upon exposure to the air or the slightest touch. About four feet of one of the tusks had been taken out up to last evening, which measured 15 inches in diameter where it joined the head. The fragments of the remains of this huge animal will afford an interesting study for the scientist, and it is Mr. Farrow's intention to donate some of them to the University in Virginia, of which State he is a native.—*Kentuckian.*

### HOW THE SPECTROSCOPE FORETELLS RAIN.

And if the air vertically above any one place becomes presently charged with more than its usual dose of such transparent watery vapor (as it easily may, by various modes and processes of nature), the spectroscope shows that fact immediately, even while the sky is still blue ; clouds soon after form, or thicken if already formed, and rain presently begins to descend.

But how does the spectroscope show to the eyes what is declared to be invisible in all ordinary optical instruments ? It is partly by its power of discriminating the differently colored rays of which white light is made up, and partly by the quality impressed on the molecules of water at their primeval creation, but only recently discovered, of stopping out certain of those rays so discriminated and placed in a rainbow-colored order by the prism and slit of the spectroscope, but transmitting others freely. Hence it is that, on looking at the light of the sky through any properly adjusted spectroscope, we see, besides the Newtonian series of colors from red to violet, and besides all the thin, dark Fraunhofer, or solar originated lines, of which it is not my object now to speak, we see, I say, in one very definite part—viz., between the orange and yellow of that row of colors, or "spectrum," as it is called—a dark, hazy band stretching across it. That is the chief band of watery vapor ; and to see it very dark, even black, do not look at a dark part of the sky or at black clouds therein, but look, rather, where the sky is brightest, fullest of light to the naked eye, and where you can see through the greatest length of such well-illumined air, at a low, rather than high, angle of altitude, and either in warm weather, or, above all, just before heavy rain-fall, when there is and must be an extra supply of watery vapor in the atmosphere. Any extreme darkness, therefore, seen in that water-vapor band beyond what is usual for the season of the year and the latitude of the place is an indication of rain-material accumulating abnormally ; while, on the other hand, any notable deficiency in the darkness of it, other circumstances being the same, gives probability of dry weather, or absence of rain for very want of material to make it ; and the band has, therefore, been called, shortly, "the rain-band." Thus, also, "rain-band spectroscopes" have been specially contructed by several most expert opticians in size so small as to be carriable in the waist-coat-pocket, but so powerful and true that a glanee of two seconds' duration through one of them suffices to tell an experienced observer the general condition of the whole atmosphere. Especially, too, of the upper parts of it, where any changes—as they take place there almost invariably earlier than below—enable such an observer to favor his friends around him with a prevision of what they are likely soon to experience.—PROFESSOR C. PIAZZI SMYTH, *in Popular Science Monthly for December.*

### A MONSTER STEEL SPRING.

On the 17th of October, there was made at Pittsburgh the largest steel spring in the world. It is the first of a series of eight, destined to act as street car motors. The initial spring was made of open hearth steel, with a carbon percentage of 055. The ingot was cast 14 x 14 inches and 7 feet long. This was rolled down to a bloom 6x4 inches and 24 feet long. To properly heat this bloom, a heating furnace 30 feet in length was built at the Superior Iron and

Steel Works, Pittsburg. The next operation, the final rolling, was the most interesting, and was only possible through the use of the Kloman "universal" mill or rolls. These had been devised by the late Andrew Kloman, and have become widely known in connection with the first successful rolling of weldless steel eyebars for structural purposes. By means of hydraulic pressure, acting through a toggle joint, an enormous pressure can be brought to bear upon the metal during its passage between the rolls, while a very quick reversal is also possible. The steel bloom referred to was rolled in this mill, in 30-feet sections, down to a length of 150 feet and 6x½ inches, and finally to a length of 310 feet and a perfectly uniform width of 6 inches and thickness of ¼ inch. Its weight was then 1,700 pounds; and to ship it, the spring was coiled in ten layers around a 4-foot pulley, the latter being given a "slow motion" as the band emerged from the heating furnace. The process of tempering and final coiling, etc., will be done in Philadelphia by the United States Spring Car Motor Company.—*Scientific American.*

